# Antibody targeted CpG for the immunotherapy of solid tumors

**DOI:** 10.1186/2051-1426-3-S2-P287

**Published:** 2015-11-04

**Authors:** Alan L Epstein, Julie Jang, Peisheng Hu, Leslie A Khawli

**Affiliations:** 1 USC Keck School of Medicine, Los Angeles, CA, USA

## Introduction

This work describes a method to improve the therapeutic effects of unmethylated CpG oligodeoxynucleotides (ODNs) analogues which can be used to stimulate the innate immune system. It is based upon the generation of novel reagents that can be used to target tumors after linkage of CpG ODNs analogues to tumor targeting monoclonal antibodies. This approach to cancer immunotherapy is novel in concept and will yield important information regarding the effectiveness of components of innate immunity in the treatment of cancer and related diseases and its ability to alter the tumor microenvironment. CpG has been used exclusively as adjuvants by injection locally at the site of interest or mixed with a tumor vaccine. For systemic diseases such as cancer, however, the local administration of CpG would limit the potential of this TLR agonist in the clinic.

## Methods

In order to generate a reagent that can be used systemically, we have linked chemically active CpG motifs analogues to tumor targeting antibodies (chTNT-3) such as those that target tumor necrosis. *In vitro* and *in vivo* studies were also performed to demonstrate that conjugation of CpG to chTNT-3 retained the immunostimulatory activity of the CpG moiety and the binding ability of the chTNT-3 antibody.

## Results

These studies validate the usefulness of this novel approach and showed that, compared with the free CpG, the chTNT-3/CpG immunoconjugate is able to stimulate the immune system *in vitro* and reduce tumor burden as a monotherapeutic agent *in vivo*. Intraperitoneal injections of chTNT-3/CpG delayed tumor growth and improved survival in both high and low immunogenic tumor models, and was not significantly different than intratumoral injections of free CpG. Compared to saline-treated mice, chTNT-3/CpG-treated mice had decreased tumor volumes by as much as 70-79 % in two diversely different tumor mouse models. Systemically delivered free CpG and CpG conjugated to an isotype control antibody did not reduce tumor burden or improve survival.

## Conclusions

These studies demonstrate the feasibility of antibody-CpG immunoconjugates for targeted therapy of innate immunity in different types of tumors and provide the foundation for near future development and clinical cancer immunotherapy evaluation.

